# Comparing i-Tree Eco Estimates of Particulate Matter Deposition with Leaf and Canopy Measurements in an Urban Mediterranean Holm Oak Forest

**DOI:** 10.1021/acs.est.0c07679

**Published:** 2021-04-28

**Authors:** Rocco Pace, Gabriele Guidolotti, Chiara Baldacchini, Emanuele Pallozzi, Rüdiger Grote, David J. Nowak, Carlo Calfapietra

**Affiliations:** † Institute of Research on Terrestrial Ecosystems (IRET), National Research Council (CNR), Porano (TR), 05010, Italy; ‡ Biophysics and Nanoscience Centre, Department of Ecological and Biological Sciences (DEB), University of Tuscia, Viterbo, 01100, Italy; § Institute of Research on Terrestrial Ecosystems (IRET), National Research Council (CNR), Monterotondo Scalo (RM), 00015, Italy; ∥ Institute of Meteorology and Climate Research, Atmospheric Environmental Research (IMK-IFU), Karlsruhe Institute of Technology (KIT), Garmisch-Partenkirchen, 82467, Germany; ⊥ USDA Forest Service, Northern Research Station, Syracuse, New York 13210, United States

**Keywords:** Air quality, PM removal, Eddy covariance, Vacuum filtration, SEM analysis, Modeling, Resuspension, Human health

## Abstract

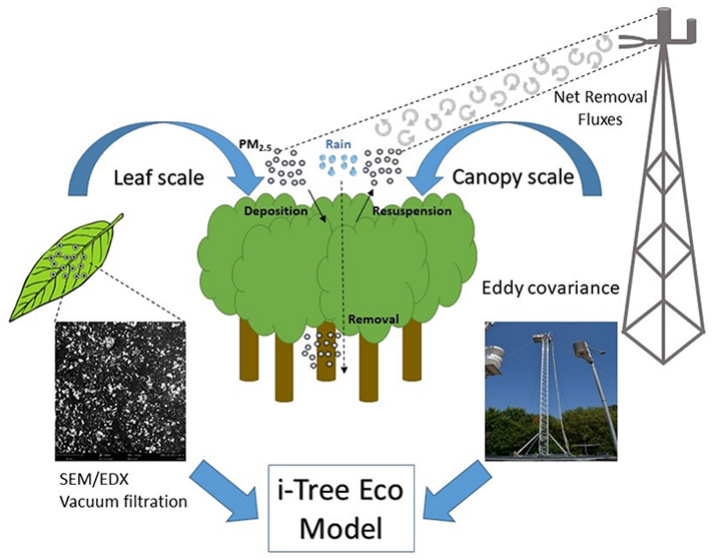

Trees and urban forests remove particulate matter (PM) from the air through the deposition of particles on the leaf surface, thus helping to improve air quality and reduce respiratory problems in urban areas. Leaf deposited PM, in turn, is either resuspended back into the atmosphere, washed off during rain events or transported to the ground with litterfall. The net amount of PM removed depends on crown and leaf characteristics, air pollution concentration, and weather conditions, such as wind speed and precipitation. Many existing deposition models, such as *i-Tree Eco*, calculate PM_2.5_ removal using a uniform deposition velocity function and resuspension rate for all tree species, which vary based on leaf area and wind speed. However, model results are seldom validated with experimental data. In this study, we compared *i-Tree Eco* calculations of PM_2.5_ deposition with fluxes determined by eddy covariance assessments (canopy scale) and particulate matter accumulated on leaves derived from measurements of vacuum/filtration technique as well as scanning electron microscopy combined with energy-dispersive X-ray spectroscopy (leaf scale). These investigations were carried out at the Capodimonte Royal Forest in Naples. Modeled and measured fluxes showed good overall agreement, demonstrating that net deposition mostly happened in the first part of the day when atmospheric PM concentration is higher, followed by high resuspension rates in the second part of the day, corresponding with increased wind speeds. The sensitivity analysis of the model parameters showed that a better representation of PM deposition fluxes could be achieved with adjusted deposition velocities. It is also likely that the standard assumption of a complete removal of particulate matter, after precipitation events that exceed the water storage capacity of the canopy (Ps), should be reconsidered to better account for specific leaf traits. These results represent the first validation of *i-Tree Eco* PM removal with experimental data and are a starting point for improving the model parametrization and the estimate of particulate matter removed by urban trees.

## Abstract 2

Cross evaluation of modeled airborne PM2.5 deposition with experimental measurements to properly estimate the contribution of urban green spaces to air quality improvement.

## Introduction

Improving air quality is a priority in many urban areas because pollution concentration often exceeds thresholds established by national or international legislation.^1^ One of the most dangerous pollutants is fine particulate matter (PM_2.5_) because tiny particles can be inhaled and affect the respiratory system.^2^ The concentration of these particles is affected by the balance between the pollutant emission, formation, and atmospheric conditions, and pollutant removal by wet and dry deposition to various surfaces. The main sources of airborne particulate matter are not only human activities (industries, households, and vehicles) but also natural ones such as wind-blown desert dust particles or sea spray aerosols.^3^

For dry deposition, vegetation represents one of the most effective sinks.^4^ To decrease the concentration of airborne particles, nature-based solutions, including an increased abundance of trees, due to their high leaf exposure surface (LAI), has been suggested as a sustainable approach for air pollution mitigation.^5,6^ However, vegetation properties as well as climatic conditions affect the efficiency of particle removal because PM is not only deposited on the vegetation surfaces but is also washed off during rain events (or transported to the ground with litterfall) and resuspended into the atmosphere.^7^ The net amount of PM removed thus depends on crown and leaf characteristics, air pollution concentration, and weather conditions, such as wind speed and precipitation.^8−10^

Consequently, relatively complex models are needed to evaluate the overall removal, which can help decision makers to optimize vegetation management and planting programs. The *i-Tree* model^7^ together with Computational Fluid Dynamics (CFD) simulations^11,12^ are the most common models to estimate PM removal from urban vegetation. These models are based on relatively coarse assumptions with only little consideration of leaf traits. For example, the *i-Tree Eco* model, which is the most commonly used urban forest model to evaluate a number of ecosystem services of urban trees,^13^ uses common deposition velocity procedures and resuspension rates for all tree species based on total leaf area and wind speed.^7^

However, the ability of tree species to capture and retain PM on leaf surfaces varies according to foliar traits^14^ such as epicuticular waxes,^15^ trichome density,^16^ and surface roughness.^17^ In addition, conifers are generally more efficient at capturing PM_2.5_ than broadleaved species^18^ due to their needle-like leaves which are smaller and more effectively arranged, resulting in a larger leaf area exposure (LAD).^19,20^ Due to these uncertainty factors,^13^ a first sensitivity study on the *i-Tree Eco* assumptions was recently carried out, suggesting the distinguishing of deposition velocities for conifers and broadleaves.^21^

Evaluation of model estimates with PM deposition data at canopy or leaf level is relatively seldom described in the literature. A good correlation was found between simulated PM_10_ deposition on tree crowns, using a CFD pollutant dispersion model (ENVI-met), and PM quantified on leaves, with Saturation Isothermal Remanent Magnetization (SIRM).^22^ Eddy covariance (EC) measurements have also been used to evaluate PM deposition models.^23,24^

In general, various approaches exist to assess different properties of leaf deposited PM, many of them based on detailed leaf assessment such as vacuum/filtration (VF) technique,^25−28^ atomic absorption spectroscopy (AAS),^29,30^ inductively coupled plasma atomic emission spectroscopy (ICP-OES),^31^ mass spectrometry (ICP-MS),^31,32^ X-ray fluorescence (XRF),^32^ scanning electron microscopy coupled with energy dispersive X-ray spectroscopy (SEM/EDX),^14,20,33^ or a combination of methods to obtain complementary information about particle size, morphology, and composition.^31,32^ These methods require leaf sampling in the field and can thus only be carried out in relatively low temporal resolution (days to weeks), which is unsuitable to detect the impact of diurnal patterns and related effects of wind speed and PM concentration on deposition and resuspension.

In contrast, the EC technique provides direct measurements of the net surface-atmosphere exchange of gases and particles.^23,34,35^ EC can operate at high temporal resolution, thus it is effective to understand flux temporal dynamics. From a spatial point of view, EC requires a homogeneous area that is difficult to meet within the urban context: these areas are typically characterized by different surface roughness^36,37^ and limited forested area, with the consequence that results can have a lower resolution and cannot be generalized.^38,39^ A single measurement point can integrate an area ranging from hundreds of square meters up to a few square kilometers, resulting in a level of uncertainty that spans from 6% in natural areas^40^ to about 12% in urban areas.^39^ The combination of measurements at leaf and ecosystem scales enables evaluation on different temporal and spatial resolution, but it has rarely been used to assess PM net exchanges.

In this study, we compared the net PM deposition flux calculated by the *i-Tree Eco* model with EC assessments within and above a Mediterranean urban forest located in the city of Naples (Italy) to evaluate the dry deposition trend over the day (canopy scale). We then used PM loads on the leaf surface measured by SEM/EDX and VF to validate the accumulation range estimated by the model (leaf scale). Furthermore, a sensitivity analysis was performed to assess the effect of different parameters on the accuracy of model evaluations using a specific deposition velocity for broadleaf trees.

The study aims to provide the first comprehensive and consistent evaluation of model assumptions for PM_2.5_ removal to properly quantify the contribution of urban trees in removing airborne particulate matter relative to different environmental boundary conditions. Finally, we discussed the pros and cons of the applied techniques and depict model deficits, also suggesting specific future improvements.

## Methods

### Study Area

The study area is the Real Bosco di Capodimonte, a Mediterranean urban forest located within the city of Naples, Italy (40.8725° N, 14.2533° E; area = 117.27 km^2^, population = 944148). Particulate matter pollution is particularly relevant in Italian cities where concentrations are higher than European standards, and the main PM sources are combustion and agriculture.^1^ In our study area, the average PM_2.5_ from 2015 to 2019 was 16.2 μg m^–3^ and the main sources of particulate matter are traffic, heating, and Saharan dusts (PM_10_) (Agenzia Regionale per la Protezione Ambientale della Regione Campania, http://www.arpacampania.it). The forest is dominated by *Quercus ilex* L. with a few large trees of *Pinus pinea* L. and some open areas of meadows mainly composed of *Trifolium* L. and *Medicago* L. The climate is typically Mediterranean, characterized by prolonged dry summer periods and mild winters, with a mean annual temperature of 16.3° and precipitation of 855 mm.^41^ At the end of June 2017, a leaf area index (LAI) of 5 was measured using two different LAI 2000 Canopy Analyzers (Li-Cor) in 5 representative areas of the forest, measuring above and below the tree canopy, respectively.

### SEM/EDX and Vacuum Filtration Measurements

Wind speed and precipitation data from January to February first, 2017 (day-of-year – DOY- 1–32) were measured at a 10 min resolution with a weather station located in the forest (Osservatorio Meteorologico Università degli Studi di Napoli Federico II, http://www.meteo.unina.it/bosco-di-capodimonte). PM_2.5_ concentrations in the same days were collected with a hourly resolution by the regional Environmental Agency ARPA Campania in two surrounding urban areas outside the park boundaries: the Astronomical Observatory (NA01:40.863643° N, 14.255496° E, about 400 m southwest) and the National Museum (NA06:40.853679° N, 14.250484° E, about 1.3 Km south).

The sampling of *Q. ilex* leaves, the dominant species in the park, was carried out on February 1, 2017 at seven different locations inside the forest that were located along the two main wind directions within an area of less than 5 ha. Only previous year leaves were selected (approximated 8 months old). The scanning electron microscope was a Phenom ProX (Phenom-World, The Netherlands) coupled with an X-ray analyzer and a charge-reduction sample holder suited for nonmetalized biological materials. Two leaves were selected from each replicate branch per tree, for a total of 28 leaves (4 per tree) used for SEM/EDX analysis, and a piece of each leaf of about 1 × 1.5 cm^2^ was fixed with the adaxial surface facing upward to the head of the carbon-based stub (PELCO Tabs, Ted Pella, Inc.).

The size and number of particles size on leaf surfaces were determined by 10 random SEM images for each sample, while EDX allowed us to obtain the elemental composition. With a combination of these data, as described in Baldacchini et al. 2019,^33^ the PM_2.5_ mass per unit leaf area (μg cm^–2^) was obtained.

For vacuum filtration, ten leaves from each replicate branch per sampling location were selected. Leaf samples were carefully shaken in a flask with 250 mL of deionized water for 5 min and then scanned to measure the leaf surface using ImageJ. The wash water was prefiltered through a 100-μm pore sieve and then dragged, by a vacuum pump, through cellulose filters with a pore size of 10–15 μm measuring the size fraction between 10 and 100 μm, then through filters with a pore size of 2–4 μm measuring the size fraction 2–10 μm, and finally, through nitrocellulose membranes for 0.2 μm measuring the size fraction 0.2–2 μm.

All filters were dried in a moisture-controlled oven for 40 min at 70 °C and placed into the balance room for 30 min for equilibriation of the humidity level, and then mass was measured at the precision of ×10^–5^ g before (T1) and after (T2) filtration. The applied filter treatment for vacuum filtration measurements of leaf deposited PM upon washing^25^ was further tested in terms of reproducibility and standardized based on comparisons with other techniques.^28,31,42^ The measured mass of PM deposited on the leaves, per each size fraction, was then estimated per unit of leaf area and divided by the total two-sided leaf area washed (μg cm^–2^). Only the PM load on the filters with the smaller pore size was used to estimate PM_2.5_ load. For additional information on the methodology, see Baldacchini et al. (2019)^33^ and Ristorini et al. (2020).^31^

### Eddy Covariance Assessments

In the summer of the same year from June 13 to September 6 (DOY 164–249), an eddy covariance flux tower conducted measurements at the site. The 26 m height tower was about 4 m higher than the mean tree height.^34^ The tower was equipped with a 3-D sonic anemometer (Windmaster Pro, Gill, UK) to measure wind speed and direction. Several fast-response analyzers including an Optical Particle Counter (OPC Multichannel Monitor, FAI Instruments, IT) measured particle sizes from 0.28 to 10 μm at a frequency of 4 Hz and logged data to a CR6 datalogger (Campbell Scientific, USA). Rain was measured with a precipitation sensor (RG100, Environmental Measurements Ltd., UK).

With the EC technique, turbulent fluxes which transport trace gases and other masses are calculated based on measurements of wind speed and compound concentrations.^43^

The basic equation of the flux calculation is
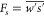
1where the vertical flux (F_S_) results from the covariance among variations around the average vertical wind speed w′ and the concentration of a scalar of interest s’ over an average period (usually half an hour). A quality control of data was applied discarding fluxes with a quality grade above 3 (0 = best quality data; 9 = worse quality data)^35^ and with a friction velocity below 0.2 m s^–1^ as suggested for the site by Guidolotti et al. (2017).^34^ For more detailed information about EC assessments, see Guidolotti et al. (2017)^34^ and Pallozzi et al. (2020).^35^

### Model Description and Simulation Setup

The PM_2.5_ deposition flux on the *Q. ilex* canopy was calculated according to the method used in the *i-Tree Eco* model^44^
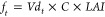
2
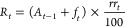
3
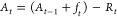
4
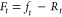
5where f_t_ is the PM_2.5_ flux at time t (g m^–2^ s^–1^), Vd_t_ is the deposition velocity at time t (m s^–1^), C is the PM_2.5_ air concentration (g m^–3^), LAI is the leaf-area index, R_t_ is the PM_2.5_ flux resuspended in the atmosphere at time t (g m^–2^ s^–1^), A_t_ is PM_2.5_ mass accumulated on leaves at time t (g m^–2^) depending on previous hour deposition as well as precipitation (A_t-1_), *rr*_t_ denotes a “resuspension class”, which is the relative amount of deposited PM_2.5_ that is resuspended at a specific wind speed at time t (%), and F_t_ is the net PM_2.5_ removal at time t after considering resuspension. The accumulated PM_2.5_ on leaves (A_t_) refers to square meters of tree cover and therefore has been rescaled by the LAI to compare it with leaf measurements.

Deposition velocities (vd_t_) and resuspension classes (rr_t_) both depend on wind speed and are defined based on the *i-Tree Eco* model standards.^7,44^ When precipitation events are higher than the maximum water storage of the canopy (Ps in mm), which is calculated according to the potential leaf water storage *plws* (0.2 mm) and LAI (Ps = plws * LAI), all PM_2.5_ accumulated on leaves is assumed to be washed off and A_t_, R_t_, and F_t_ are set to 0.^44^

Additional simulations have been carried out using the deposition velocities suggested recently by Pace and Grote (2020)^21^ for broadleaved trees (vds)
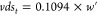
6where w′ (m s^–1^) is the wind speed at time t.

The sensitivity of the model parametrization was carried out considering a factor of 2 and 3 for the potential leaf water storage, deposition velocity, resuspension classes, and the leaf washing after rainfall events that exceed the maximum water storage of the canopy (Table 1). Furthermore, the combined effect of parameters (combo) with factors 2 and 3 was evaluated. The impact of the parameter variations to deposition and cumulative flux was assessed using a multiple comparison of means (Turkey’s HSD test).

**Table 1:** Model Parameter Modification to Assess the Deposition Flux Sensitivity

**Parameter**	**Standard**	**Factor 2**	**Factor 3**
Potential leaf storage	0.2	0.4	0.6
Deposition velocity	0.1094	0.2188	0.3282
Resuspension classes	1.00	0.5	0.33
Leaf washing	100%	50%	33%

Model simulations were performed during two different periods in 2017: DOY 1–32 for the comparison of simulated accumulated deposition with leaf measurements of PM accumulated on leaves^33^ (using hourly wind speed, precipitation, and PM_2.5_ measured at local weather stations as previously described) and DOY 164–249 for the comparison of deposition flux with EC assessments^35^ (using half-hour wind speed, precipitation, and PM_2.5_ measured at the tower).

## Results

### PM Concentrations, Wind Speed, and Precipitation

**Figure 1:**
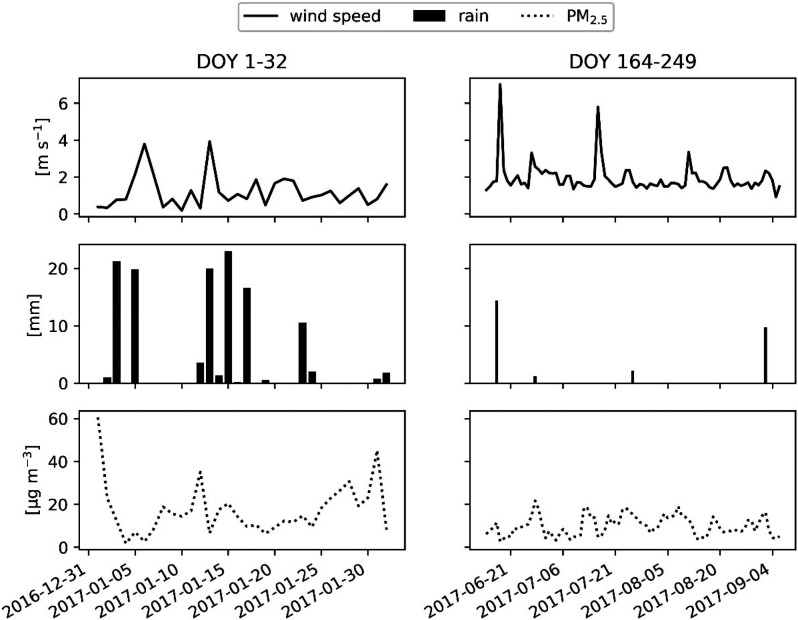
Wind speed, precipitation, and PM_2.5_ concentration throughout the two measurement campaigns. Particulate matter data are reported for the period DOY 1–32 up to the leaf sampling day (February 1st).

The two periods analyzed showed differences in wind speed, precipitation, and PM_2.5_ concentrations (Figure 1). In particular, the wind speed recorded from the eddy covariance station (DOY 164–249) is slightly greater due to the height of the tower (26 m) compared to the measurements in winter (DOY 1–32) from the local weather station (≈15 m). Precipitation is considerably lower, and intense rainfall events are much less pronounced during the summer (DOY 164–249) compared to January (DOY 1–32), which is typical of the Mediterranean climate. The particulate matter concentration is also higher during the winter (DOY 1–32) due to residential heating as well as fireworks on the first day of the year. The meteorological data obtained by the two measurement systems (EC tower and the local weather station) have been compared to demonstrate that both could be used to simulate the deposition regime during the period of DOY 164–249 (SI Figure S1–3). For this time period, PM_2.5_ concentrations are in the same order of magnitude at both places and precipitation events are almost the same. Wind speed data have a similar trend and magnitude, with larger outliers obtained with EC measurements, likely due to the greater height of the tower in comparison with the weather station.

### Model vs PM_2.5_ Leaf Accumulation

**Figure 2:**
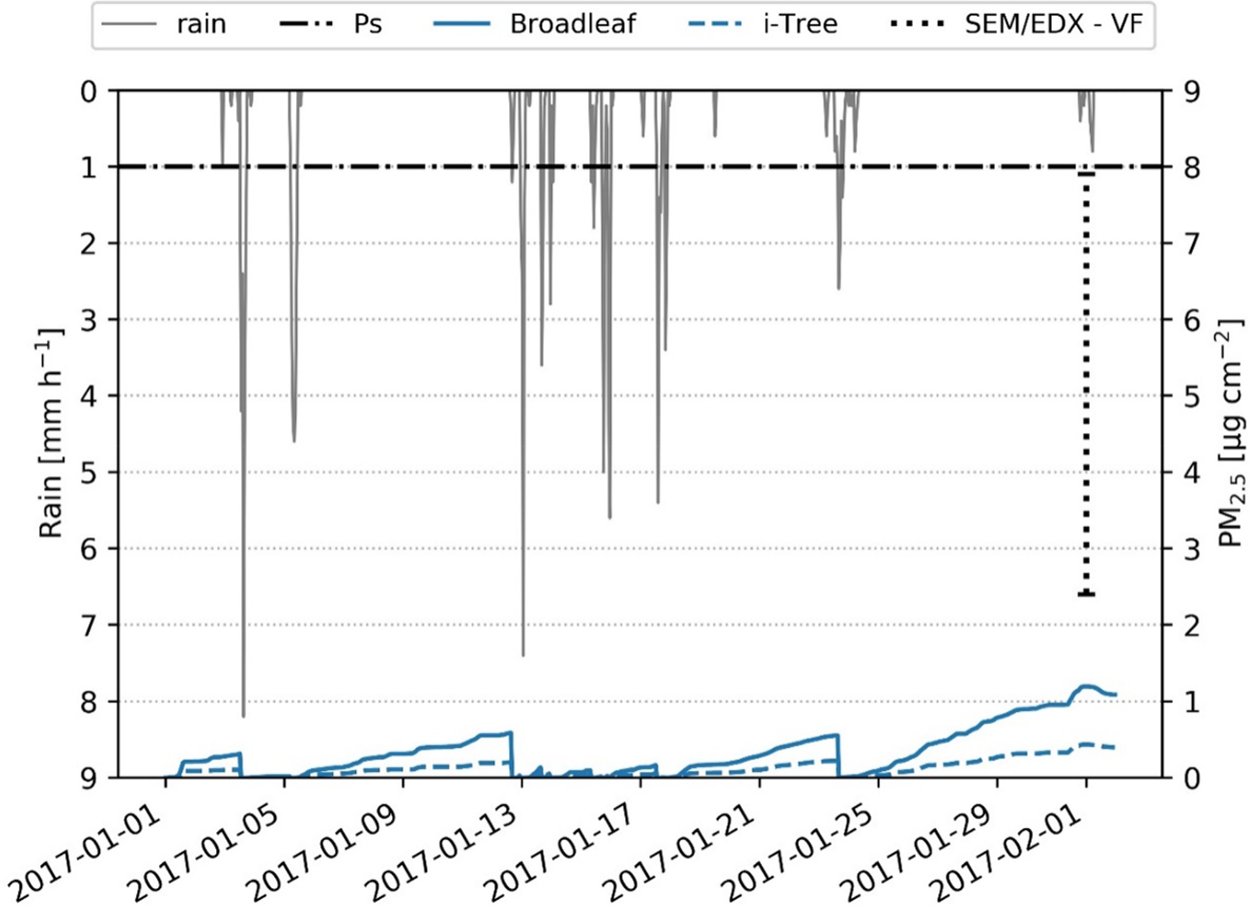
Modeled cumulative PM_2.5_ (A_t_) calculated according to the i-Tree Eco standard parametrization (i-Tree) and broadleaf specific deposition velocity (Broadleaf), compared with leaf measurements of the PM_2.5_ load by SEM/EDX and vacuum filtration (VF), on leaves collected on February 1, 2017 (min = 2.4; max = 7.9 μg cm^–2^). Precipitation events above the maximum water storage of the canopy (Ps) wash off leaves and set the cumulative flux to 0.

Both the VF and the SEM/EDX methodologies resulted in similar estimates of average PM_2.5_ mass per unit leaf area (Table 2). The modeled accumulated PM_2.5_ mass is from 6 to around 20 times lower, based on the *i-Tree Eco* parametrization (0.4 μg cm^–2^), and from about 2.2 to 7.2 times lower with the broadleaf specific deposition velocity (1.1 μg cm^–2^), in comparison to the range of values indicated by the two measurement methods (min = 2.4; max = 7.9 μg cm^–2^) (Figure 2).

**Table 2:** PM_2.5_ Mass Per Unit Leaf Area Measured by SEM/EDX and Vacuum Filtration (VF) on February 1, 2017

**PM**_**2.5**_**(μg cm**^**-2**^**)**	**MIN**	**MEAN**	**MAX**
SEM/EDX	2.4 ± 0.4	4.7 ± 1.0	7.9 ± 1.0
VF	3.0 ± 1.0	4.6 ± 0.8	6.4 ± 0.2

The SEM/EDX analysis was not able to distinguish coagulated particles from PM_10_ by automated image grain analysis, and thus the total PM_2.5_ load value might be underestimated. However, results show a similar average PM_2.5_ mass with respect to VF (Table 2), where coagulated particles are expected to be disaggregated, confirming the reliability of the methodology for PM accumulation on leaves.

A period of 30 days was considered to evaluate the model deposition calculations up to the leaf sampling date. However, the model’s ability to represent deposition is evaluated for the last week of January only, since according to the model’s internal assumptions, a high-precipitation event on January 23rd completely washed off PM from leaves (Figure 2).

### Model vs Eddy Covariance Diurnal Fluxes

**Figure 3:**
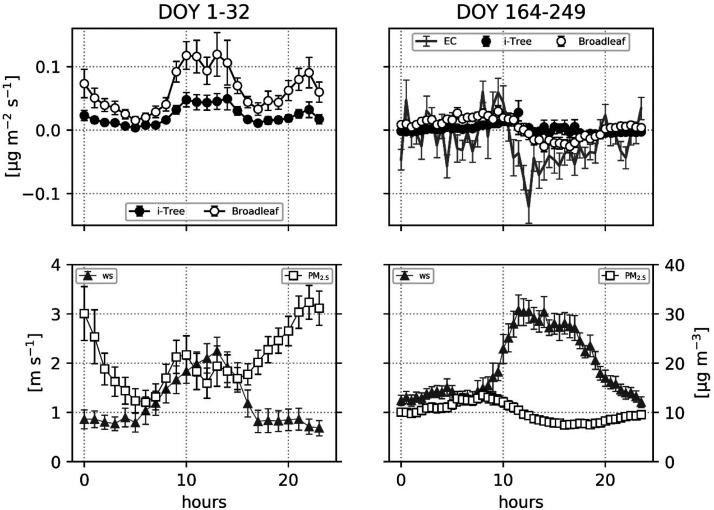
Top left: Hourly average net flux throughout the day (DOY 1–32) modeled using the i-Tree Eco standard parametrization (i-Tree) and the specific parametrization for broadleaved species (Broadleaf). Bottom left: Hourly average wind speed (ws) and particulate matter concentration (PM_2.5_) throughout the day during the same period. Top right: Half-hourly average net flux (DOY 164–249) measured by the eddy covariance (EC) and simulated fluxes using either the i-Tree Eco standard parametrization (i-Tree) or the specific parametrization for broadleaved species (Broadleaf). Bottom right: Half-hourly average wind speed (ws) and particulate matter concentration (PM_2.5_) throughout the day during the same period.

The EC in summer (DOY 164–249) indicates an average diurnal flux that is characterized by a small deposition of PM_2.5_ in the first part of the day until 10 a.m., followed by a high resuspension (release of particles back into the atmosphere) likely caused by the increase in wind speed and a decrease in airborne particle concentration that results in a negative net flux deposition (Figure 3). The higher PM concentration in the morning is related to both increased vehicular traffic during these hours along with an accumulation of pollutants during the night, which results from more stable atmospheric conditions and reduced turbulent exchange.^35^

The modeled flux with the *i-Tree Eco* parametrization shows the same range of particle deposition as determined by the EC flux, but results are less sensitive to wind speed and particulate matter variations. The maximum deposition rate using the *i-Tree Eco* parametrization is calculated for midday, when wind speed is highest, which is a bit later than indicated by the measurements. The characteristic of the model to simulate a positive net flux for PM during high wind speed periods despite simultaneously occurring high resuspension rates has already been shown by Pace and Grote 2020^21^, at least as long occasional precipitation events are reducing the accumulated PM load.

Overall, the high resuspension is better reflected by the specific broadleaf-parametrization than the standard one, resulting in an overall better fit to the trend measured with EC.

In comparison to that of summer (DOY 164–249), the simulated daily average particle deposition in winter (DOY 1–32) is much larger, predominantly due to higher pollution concentrations. During winter, resuspension processes are not dominant during any time of the day. This pattern is different in the summer period, where lower pollutant concentration and higher wind speed lead to high (measurements) or moderate (simulations) net resuspension fluxes during midday or early afternoon, respectively. The differences between simulation results and measurements may indicate either a still too small sensitivity of resuspension to wind speed or, more likely, an underestimation of the canopy particle storage (Figure 2), which limits the potential resuspension of particles.^7,21^

### Sensitivity Analysis to Model Parametrization

**Figure 4:**
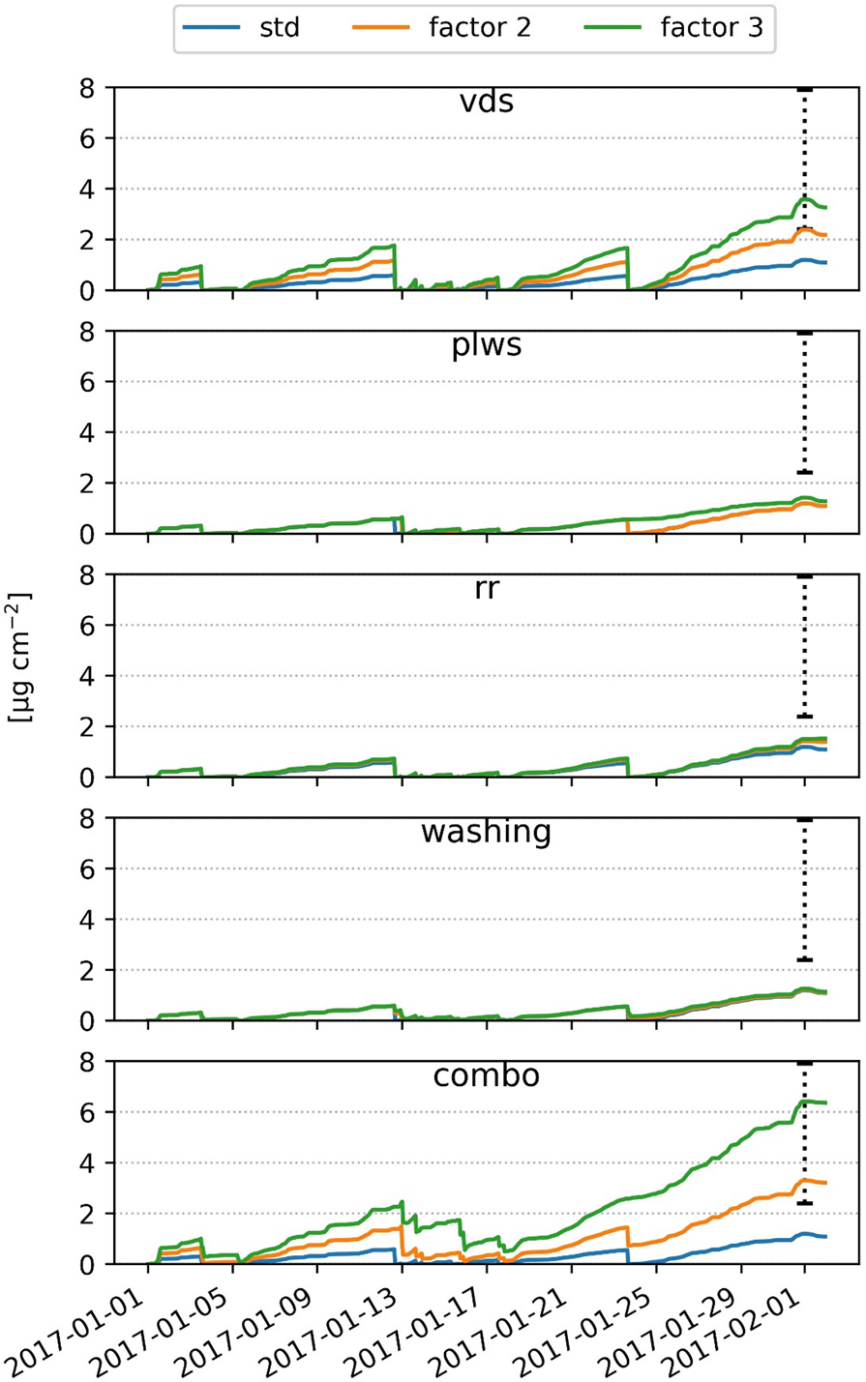
Sensitivity analysis of the modeled PM_2.5_ accumulation on leaves (DOY 1–32) to the deposition velocity (vds), potential leaf water storage (plws), resuspension classes (rr), leaf washing (washing), and combining the different parametrization (combo). The dashed line indicates the leaf PM_2.5_ load range measured with SEM/EDX and VF collected on February 1, 2017 (min = 2.4; max = 7.9 μg cm^–2^).

By increasing the deposition velocity (vds) by at least a factor of 2, the PM_2.5_ accumulation estimated by the model falls within the range measured by SEM/EDX and VF (Figure 4). Model simulations are less sensitive to the variation of other parameters such as plws (potential leaf water storage), rr (resuspension rate), and washing (leaf washing). However, the combined effect of all parameters (combo) results in a better fit to the average of leaf measurements than vds changes alone. In particular, the higher maximum water storage of the canopy (Ps) which depends on plws, the reduced leaf washing after rainfall events (washing), and a lower resuspension rate (rr) allow a larger deposition of PM_2.5_ on leaves. The multiple comparison of means (Tukey HSD) shows significant differences with the “standard” simulation only for the “washing” and “combo” run (SI Table S1).

**Figure 5:**
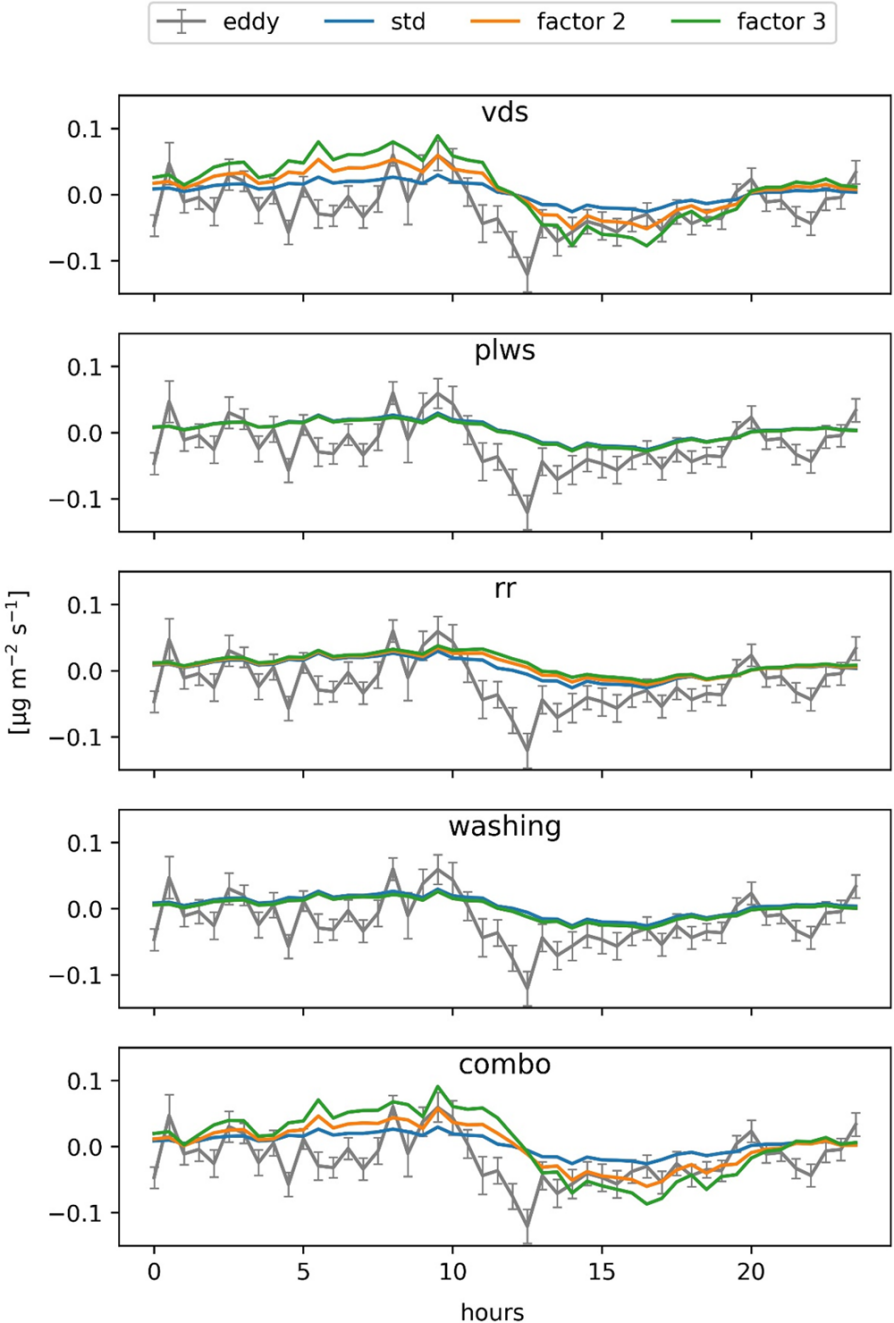
Sensitivity analysis of the modeled PM_2.5_ net flux to the deposition velocity (vds), potential leaf water storage (plws), resuspension classes (rr), leaf washing (washing), and combining the different parametrization (combo) compared with the eddy covariance flux (DOY 164–249).

The high sensitivity of the model to deposition velocity, compared to the other parameters, is also apparent from the comparison of the modeled PM_2.5_ net flux with the EC assessment (Figure 5). In particular, an increase by a factor of 2 better matches the deposition peaks in the first part of the day as well as the high resuspension rates during the afternoon. Since the sensitivity of net pollution removal to changes of parameters other than vds is very small, the combined effect of all the parameters (combo) is very similar to the effect on vds changes with a slight delay in the negative flux trend due to the lower resuspension rates (rr). The multiple comparison of means (Tukey HSD) shows significant differences with the “standard” simulation only in compariosn with the change in “vds” by a factor of 3 (SI Table S2).

## Discussion

It is known that PM removal from urban trees depends on the morphological properties of the vegetation, the seasonal changes in leaf development,^45^ and environmental parameters including PM concentration, wind speed, and precipitation rate.^46,47^ The Mediterranean climate is characterized by long periods of summer drought when PM accumulated on the leaves is not washed off by rain but may be exposed to wind resuspension.^48^ Here, we show that periods of high resuspension occur, generating a negative net flux, especially in the second part of the day (Figure 3). This pattern was particularly evident when analyzing EC measurements in the summer period (DOY 164–249; Figure 3), compared to the modeled net flux in the winter period (DOY 1–32; Figure 3), where the trend follows the development of wind speed with a higher deposition at mid-day hours. Another EC study of PM deposition on a *Q. ilex* L. forest in Rome, mainly carried out in summer, also showed the same trend of a high resuspension in the middle of the day.^23^ These results have been also confirmed from modeling simulations by Nowak et al. (2013)^7^ and Pace and Grote (2020),^21^ showing an increase in particle resuspension with increased wind speed. A different seasonal pattern in winter is also visible from the EC assessments carried out in February 2018 at the same site by Pallozzi et al. (2020)^35^ where, on the contrary, the deposition mainly occurs in the central hours of the day. Performing a model simulation for the same period and location, we obtained a net flux in the same range as determined in the above-mentioned study^35^ (SI Figure S4). In particular, model- and EC results are similar during the deposition phase at midday. However, simulations diverge from measurements for the early and late hours of the day, where the model tends to calculate deposition while net resuspension has been measured with the EC method.

A modeling concept that considers the most important in- and out-flows in mechanistic dependency on wind speed could represent the range of the net removal flux (between −0.1 and +0.1 μg m^–2^ s^–1^) and pattern of the measurements, although the high resuspension rates could only be simulated when velocity parameters were considerably larger than originally considered (Figure 3). This finding is, however, to be treated with caution. Since the measured outflow of particles (leading to a negative net removal rate) is considerably high, it can be hypothesized that particles may not only originate from previous leaf deposition but also from other sources (e.g., soil), as the footprint defined by EC is relatively heterogeneous (forest, meadow, building).^34,35^ Regarding our EC station, Pallozzi et al. (2020)^35^ estimated that on average up to the 80% of the footprint was within the park boundaries at both day and night time.

Only a few studies have investigated the role of urban landscapes on EC fluxes. A specific split footprint approach was implemented for PM by Järvi et al. (2009)^49^ in a heterogeneous area of Helsinki, revealing a smaller impact of vegetated areas than of unvegetated ones on PM fluxes. However, a reliable evaluation of the effect of vegetated and nonvegetated areas on fluxes requires the presence of an EC tower network.^37,50^ Furthermore, it should be noted that compared to gas exchange, which includes a larger data set of net flux measurements, the high-quality control applied for particles discarded about 60% of the half-hour data, resulting in a less robust data set^35^ that did not allow for the comparison of modeled data with the cumulated EC flux data.

Overall, the model calculation, using a specific vd for broadleaf trees based on wind speed (eq 6), performed better compared to the *i-Tree Eco* parametrization, which uses a specific vd for different wind speed classes.^7^ The latter is considerably less sensitive to wind speed, resulting in a smaller deposition flux that is almost offset by resuspension. In effect, the *i-Tree* parametrization leads to a slightly declining net deposition flux after midday which is not in accordance with measurements (Figure 3). The current parametrization could be improved by increasing the vd (Figures 4, 5). In fact, a higher vd is also supported from other model approaches and experimental measurements. For example, PM_2.5_ deposition simulations for the city of Leicester (UK), assessed with a Computational Fluid Dynamics model, used a vd of 0.64 cm s^–1^ which is about 3-fold the value implemented in *i-Tree*.^11^ Sun et al. (2014)^51^ also measured an average vd above a deciduous forest in spring of about 1 cm s^–1^ during the day. An improvement in model parametrization is thus required, in particular with regard to the deposition velocity (SI Figure S5), which allows not only a better estimation of leaf accumulation (Figure 4) but also a better agreement with the net deposition flux (Figure 5).

Another model uncertainty is related to the amount of PM removed by precipitation. Xu et al. (2017)^10^ found that PM wash-off rates increase with cumulative precipitation up to a maximum amount of 12.5 mm of rain, removing 51 to 70% of PM accumulated on leaves, with a small amount of PM still retained on the leaf surface. Washing rate varies with precipitation regime and leaf retention properties.^52^ PM removal is stronger with low intensity rainfall at smooth leaf surfaces, while rough leaf surfaces release more PM under short-duration, high-intensity events.^53^ Smooth and waxy surfaces cannot hold as many particles per unit leaf area as leaves with rough surfaces.^54^ Furthermore, leaves with trichomes and wax accumulations at the surface are known to strongly hold on to PM, often keeping a certain percentage of particles, particularly smaller particles, regardless of precipitation intensity.^47,55,56^

In our study, several precipitations events occurred before the leaf sampling (DOY 1–32, Figure 1) and based on the current parametrization in *i-Tree Eco* (standard) the last event on January 23rd, which was above the maximum canopy water storage (1 mm), washed off all particulate matter from leaves (A_t_ = 0) (Figure 2). We therefore hypothesize that the underestimation of PM accumulation by the model, compared with VF and SEM/EDX measurements (Figure 2), may partially result from not considering older particles that are tightly bound to leaves or particles that were on the leaves prior to DOY 1.

The “combo” run in the sensitivity analysis of the model parametrization (Figure 4) showed that by increasing the water storage of the canopy (PS), reducing the percentage of leaf washing after rainfall events above the threshold, as well as reducing the resuspension rate, tree leaves accumulate more PM_2.5_ and attain values closer to the range measured by leaf analysis. The quantity of particles on the leaves that is transported to the ground by rainfall is important for the estimation of the total amount of PM removed by trees. If we compare the results of the “standard” parametrization, where all the amount of PM accumulated on leaves is washed off by rain events above Ps, with the “combo” run considering a factor 3 where only 33% of PM is removed (Figure 4), the difference in overall PM removal is relatively small (standard = 0.16 g m^–2^ – combo = 0.21 g m^–2^). The reason for this minimal difference is that although in the case of standard parametrization 100% of PM is removed in one event, the amount of PM accumulated on the leaves is much lower compared to the “combo” simulation.

Both model parametrizations underestimate the PM that accumulates on the leaves compared to the techniques carried out at leaf level (Figure 2). The VF and SEM/EDX showed a good agreement in the measurement of fine PM load (about 5 μg cm^–2^ on average, in both cases; Table 2), a value that is in accordance with other experiments on broadleaves (about 5 μg cm^–2^^18,26,27,57^). In another study that also used the VF technique, a similar amount of PM_2.5_ (on average over four sites that represented a rural-urban gradient 4.2 ± 0.8 μg cm^–2^) was found by VF on leaves of *Q. ilex* in January, but highest values were recorded in August especially in some sites (on average 13.4 ± 1.9 μg cm^–2^).^28^ These results show that site and weather conditions are important for determining the actual accumulation of PM and that measurements during a specific time-period are not representative for the whole year. However, they may still be of use for the evaluation of model processes as long as driving forces such as weather conditions are correctly considered.

The fraction of particles that accumulates on the leaf surface depends on species-specific properties and increases with the abundance of trichomes,^16,18,58^ epicuticular waxes,^15,25,26^ and surface roughness.^17^ An accumulation index has been recently developed considering a number of leaf properties analyzed with a microscope, which will help to rank the various species and to optimize those planting programs aimed at maximizing PM removal.^14^*Q. ilex* is a common urban tree in Mediterranean cities,^59,60^ and it is an evergreen species with a higher LAI than most other broadleaves, which makes it particularly suitable for the accumulation of particulate matter on leaves^28,61^ and less subject to seasonal variation related to leaf development.^45^ Thanks to the presence of trichomes and specific leaf area, it was recently classified as one of the most effective particle accumulators of urban plant species.^16^ Furthermore, the presence of epicuticular waxes on *Q. ilex* leaves and a good retention capacity enhance the accumulation of fine particles and the adsorption of lipophilic organic pollutants.^28,61^

All these factors may partially justify the underestimation observed in the model calculation of leaf deposited PM amount (Figure 2). Specific leaf morphological traits may hold PM much tighter,^45,61^ demanding more water for washing^10^ and decreasing the amount of PM which may resuspend.^62^ A tight adherence of particles may result from a larger amount of leaf-encapsulated particle.^45^ This is not included in the present model but deserves more interest in future model development.

Although this investigation does not provide an overview about different species responses, it is likely from the current study and literature that a species-specific parametrization could improve the accuracy of model estimates. For example, distinguishing specific deposition velocities for conifers and broadleaves,^21^ considering the influence of various foliage traits on resuspension rates^12,63^ and leaf washing,^53^ could help improve model estimates. Also, the *i-Tree Eco* model uses a big-leaf approach for PM_2.5_ estimates and the calculation of PM removal might be improved using a multilayered canopy distribution,^64^ which could allow for a distinction of leaves exposed to specific wind speeds and intercepted precipitation. In fact, rainfall and wind intensities vary within the tree canopy, with upper-canopy layers more exposed to rain washing and resuspension of particles by wind in comparison to lower canopy layers.

While several studies across the world focus on improving the estimates of PM removal by urban vegetation, we provide here, for the first time, a comparison of simulated PM_2.5_ deposition using the methodology implemented in *i-Tree Eco*, the most commonly used model in urban forestry,^13^ with different field measurement techniques of canopies (EC) and leaves (VF and SEM/EDX).

In general, the simulations were able to adequately represent the PM deposition on an urban forest, indicated by similar magnitudes and dynamics as obtained with measurements at different scales (leaf, canopy, forest). However, our sensitivity analysis indicated that the current parametrization of *i-Tree Eco* is suboptimal for the specific case investigated here. In particular, incorporating the impact of leaf traits that determine parameters of particulate matter accumulation and resuspension, which directly affect the deposition velocity and the leaf washing process, would likely improve model estimates of PM removal by local urban forests.

In addition, longer-term studies with more frequent determination of PM_2.5_ accumulation would be beneficial to determine potential accumulation limits or a dependence of resuspension from PM storages on leaves. Since the importance of leaf properties is highlighted in the literature, future research should expand the investigation of species-specific leaf impacts on PM vd, wash off, and resuspension rates to aid in model parametrization.

## Supporting Information Available

The Supporting Information provides additional information on the representativeness of local weather and pollution stations compared to data measured by the EC tower, results of the multiple comparison of accumulated and net flux means (Turkey’s HSD) performing model simulations with a change in parameters, a comparison between the model and EC assessments in February 2018, and the sensitivity analysis of the deposition velocity considering a modification of parameters compared with EC results. The Supporting Information is available free of charge at https://pubs.acs.org/doi/10.1021/acs.est.0c07679.

Figures S1−S5; Tables S1 and S2 (PDF)
