# Supplementary material for: Comparing i-Tree Eco Estimates of Particulate Matter Deposition with Leaf and Canopy Measurements in an Urban Mediterranean Holm Oak Forest
Source: Environ Sci Technol. 2021 Apr 28;55(10):6613–22. doi: 10.1021/acs.est.0c07679 (PMC9282645; doi:10.1021/acs.est.0c07679)
Supplement: Supplementary file 1 — es0c07679_si_001.pdf [file es0c07679_si_001.pdf]

Comparing i-Tree Eco estimates of particulate matter deposition with leaf and canopy measurements in an urban Mediterranean holm oak forest

Rocco Pace[1], Gabriele Guidolotti[1*], Chiara Baldacchini[1-2], Emanuele Pallozzi[3], Rüdiger Grote[4], David J. Nowak[5], Carlo Calfapietra[1]

[1] Institute of Research on Terrestrial Ecosystems (IRET), National Research Council (CNR), Porano (TR), Italy
[2] Biophysics and Nanoscience Centre, Department of Ecological and Biological Sciences (DEB), University of Tuscia, Viterbo, Italy
[3] Institute of Research on Terrestrial Ecosystems (IRET), National Research Council (CNR), Monterotondo Scalo (RM), Italy
[4] Institute of Meteorology and Climate Research, Atmospheric Environmental Research (IMK-IFU), Karlsruhe Institute of Technology (KIT), Garmisch-Partenkirchen, Germany
[5] USDA Forest Service, Northern Research Station, Syracuse, NY, USA

PAGES: S1-S7

FIGURES: S1-S5

TABLES: S1-S2

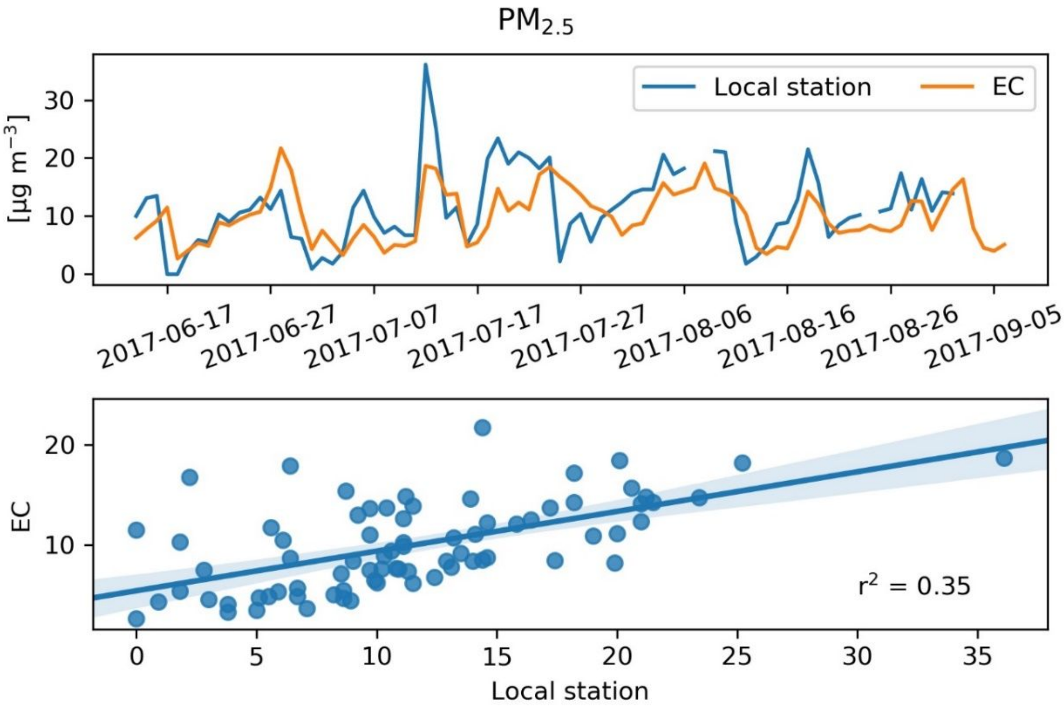

Figure S1. *Comparison of* $PM_{2.5}$ *concentration measured by the pollution station in Naples (Local station) and the EC tower (EC) during the period DOY 164-249.*

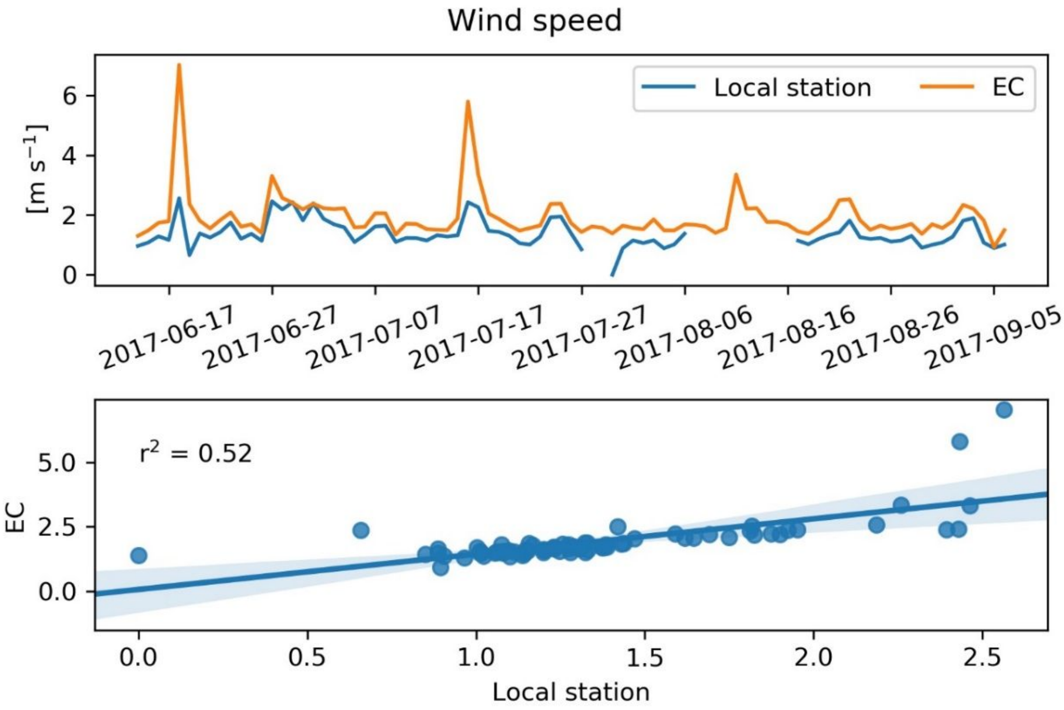

Figure S2. *Comparison of wind speed measured by the weather station located at the Capodimonte Royal Forest in Naples (local station) and EC tower (EC) during the period DOY 164-249.*

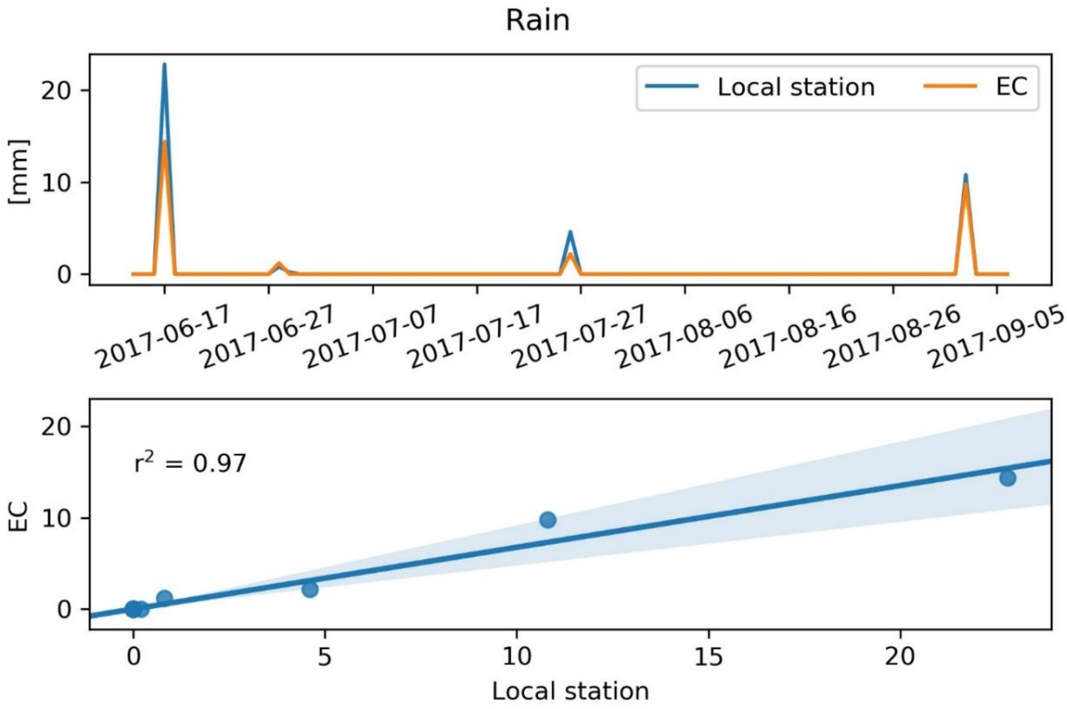

Figure S3. *Comparison of precipitation measured by the weather station located at the Capodimonte Royal Forest in Naples (local station) and EC tower (EC) during the period DOY 164-249.*

Table S1. *Results of the multiple comparison of accumulated flux means (Turkey's HSD) performing model simulations with a modification of parameters (plws, vds, rr, washing). In bold, the standard simulation compared to parameter variation with factor 2 and 3.*

ACCUMFLUX: Multiple Comparison of Means - Tukey HSD,FWER=0.05

group1	group2	meandiff	lower	upper	reject
combo2	combo3	1.1459	1.0479	1.2439	True
combo2	plws2	-0.9952	-1.0932	-0.8972	True
combo2	plws3	-0.9942	-1.0922	-0.8961	True
combo2	rr2	-0.9948	-1.0928	-0.8968	True
combo2	rr3	-0.9946	-1.0926	-0.8966	True
**combo2**	**std**	**-0.9986**	**-1.0966**	**-0.9006**	**True**
combo2	vds2	-0.992	-1.09	-0.894	True
combo2	vds3	-0.9887	-1.0867	-0.8907	True
combo2	wash2	-0.6484	-0.7464	-0.5503	True
combo2	wash3	-0.6214	-0.7194	-0.5234	True
combo3	plws2	-2.1411	-2.2391	-2.0431	True
combo3	plws3	-2.1401	-2.2381	-2.042	True
combo3	rr2	-2.1407	-2.2387	-2.0427	True
combo3	rr3	-2.1405	-2.2385	-2.0425	True
**combo3**	**std**	**-2.1445**	**-2.2425**	**-2.0465**	**True**
combo3	vds2	-2.1379	-2.2359	-2.0399	True

combo3	vds3	-2.1346	-2.2326	-2.0366	True
combo3	wash2	-1.7943	-1.8923	-1.6963	True
combo3	wash3	-1.7673	-1.8653	-1.6693	True
plws2	plws3	0.001	-0.097	0.0991	False
plws2	rr2	0.0004	-0.0976	0.0984	False
plws2	rr3	0.0006	-0.0974	0.0986	False
**plws2**	**std**	**-0.0034**	**-0.1014**	**0.0946**	**False**
plws2	vds2	0.0032	-0.0948	0.1012	False
plws2	vds3	0.0065	-0.0915	0.1045	False
plws2	wash2	0.3468	0.2488	0.4449	True
plws2	wash3	0.3738	0.2758	0.4718	True
plws3	rr2	-0.0007	-0.0987	0.0973	False
plws3	rr3	-0.0005	-0.0985	0.0975	False
**plws3**	**std**	**-0.0045**	**-0.1025**	**0.0936**	**False**
plws3	vds2	0.0021	-0.0959	0.1001	False
plws3	vds3	0.0054	-0.0926	0.1034	False
plws3	wash2	0.3458	0.2478	0.4438	True
plws3	wash3	0.3727	0.2747	0.4707	True
rr2	rr3	0.0002	-0.0978	0.0982	False
**rr2**	**std**	**-0.0038**	**-0.1018**	**0.0942**	**False**
rr2	vds2	0.0028	-0.0952	0.1008	False
rr2	vds3	0.0061	-0.0919	0.1041	False
rr2	wash2	0.3465	0.2484	0.4445	True
rr2	wash3	0.3734	0.2754	0.4714	True
**rr3**	**std**	**-0.004**	**-0.102**	**0.094**	**False**
rr3	vds2	0.0026	-0.0954	0.1006	False
rr3	vds3	0.0059	-0.0921	0.1039	False
rr3	wash2	0.3463	0.2483	0.4443	True
rr3	wash3	0.3732	0.2752	0.4712	True
**std**	**vds2**	**0.0066**	**-0.0914**	**0.1046**	**False**
**std**	**vds3**	**0.0099**	**-0.0881**	**0.1079**	**False**
**std**	**wash2**	**0.3503**	**0.2522**	**0.4483**	**True**
**std**	**wash3**	**0.3772**	**0.2792**	**0.4752**	**True**
vds2	vds3	0.0033	-0.0947	0.1013	False
vds2	wash2	0.3437	0.2456	0.4417	True
vds2	wash3	0.3706	0.2726	0.4686	True
vds3	wash2	0.3404	0.2424	0.4384	True
vds3	wash3	0.3673	0.2693	0.4653	True
wash2	wash3	0.0269	-0.0711	0.125	False
------	--------	---------	-------	--------	-------

Table S2. *Results of the multiple comparison of net flux means (Turkey's HSD) performing model simulations with a modification of parameters (plws, vds, rr, washing). In bold, the standard simulation compared to parameter variation with factor 2 and 3.*

NET FLUX: Multiple Comparison of Means - Tukey HSD,FWER=0.05

group1	group2	meandiff	lower	upper	reject
combo2	combo3	0.0034	-0.0034	0.0103	False
combo2	plws2	0.0005	-0.0063	0.0074	False
combo2	plws3	0.0005	-0.0063	0.0074	False
combo2	rr2	0.0055	-0.0013	0.0124	False
combo2	rr3	0.0085	0.0017	0.0154	True
**combo2**	**std**	**0.002**	**-0.0049**	**0.0088**	**False**
combo2	vds2	0.006	-0.0008	0.0128	False
combo2	vds3	0.01	0.0032	0.0168	True
combo2	wash2	-0.0017	-0.0085	0.0052	False
combo2	wash3	-0.0017	-0.0085	0.0052	False
combo3	plws2	-0.0029	-0.0097	0.0039	False
combo3	plws3	-0.0029	-0.0097	0.0039	False
combo3	rr2	0.0021	-0.0047	0.0089	False
combo3	rr3	0.0051	-0.0017	0.0119	False
**combo3**	**std**	**-0.0015**	**-0.0083**	**0.0054**	**False**
combo3	vds2	0.0026	-0.0043	0.0094	False
combo3	vds3	0.0066	-0.0003	0.0134	False
combo3	wash2	-0.0051	-0.0119	0.0017	False
combo3	wash3	-0.0051	-0.0119	0.0017	False
plws2	plws3	0	-0.0068	0.0068	False
plws2	rr2	0.005	-0.0018	0.0119	False
plws2	rr3	0.008	0.0012	0.0148	True
**plws2**	**std**	**0.0015**	**-0.0054**	**0.0083**	**False**
plws2	vds2	0.0055	-0.0014	0.0123	False
plws2	vds3	0.0095	0.0026	0.0163	True
plws2	wash2	-0.0022	-0.009	0.0047	False
plws2	wash3	-0.0022	-0.009	0.0047	False
plws3	rr2	0.005	-0.0018	0.0119	False
plws3	rr3	0.008	0.0012	0.0148	True
**plws3**	**std**	**0.0015**	**-0.0054**	**0.0083**	**False**
plws3	vds2	0.0055	-0.0014	0.0123	False
plws3	vds3	0.0095	0.0026	0.0163	True
plws3	wash2	-0.0022	-0.009	0.0047	False
plws3	wash3	-0.0022	-0.009	0.0047	False
rr2	rr3	0.003	-0.0039	0.0098	False
**rr2**	**std**	**-0.0036**	**-0.0104**	**0.0033**	**False**
rr2	vds2	0.0004	-0.0064	0.0073	False
rr2	vds3	0.0045	-0.0024	0.0113	False
rr2	wash2	-0.0072	-0.014	-0.0004	True
rr2	wash3	-0.0072	-0.014	-0.0004	True
**rr3**	**std**	**-0.0065**	**-0.0134**	**0.0003**	**False**
rr3	vds2	-0.0025	-0.0094	0.0043	False

rr3	vds3	0.0015	-0.0054	0.0083	False
rr3	wash2	-0.0102	-0.017	-0.0033	True
rr3	wash3	-0.0102	-0.017	-0.0033	True
**std**	**vds2**	**0.004**	**-0.0028**	**0.0108**	**False**
**std**	**vds3**	**0.008**	**0.0012**	**0.0149**	**True**
**std**	**wash2**	**-0.0036**	**-0.0105**	**0.0032**	**False**
**std**	**wash3**	**-0.0036**	**-0.0105**	**0.0032**	**False**
vds2	vds3	0.004	-0.0028	0.0108	False
vds2	wash2	-0.0076	-0.0145	-0.0008	True
vds2	wash3	-0.0076	-0.0145	-0.0008	True
vds3	wash2	-0.0117	-0.0185	-0.0048	True
vds3	wash3	-0.0117	-0.0185	-0.0048	True
wash2	wash3	0	-0.0068	0.0068	False
------	-------	----------	-------	--------	-------

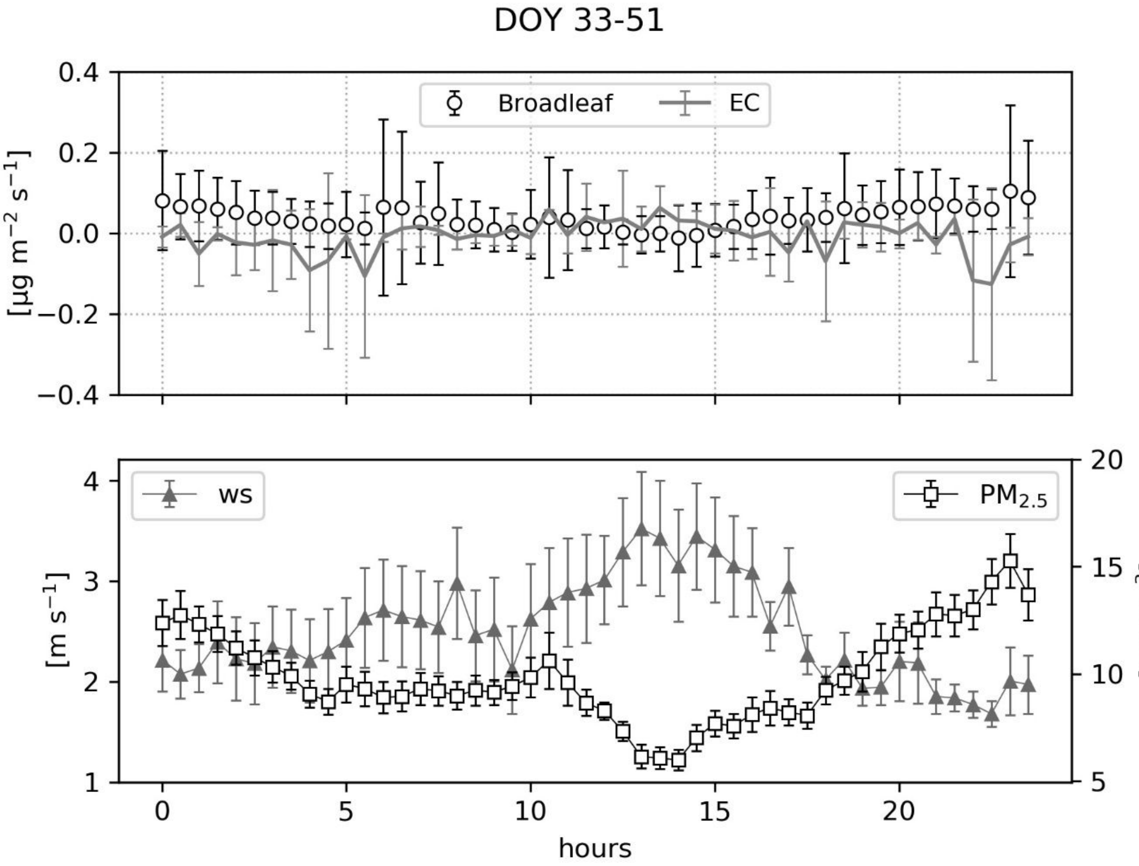

Figure S4. *Top: the average daily net flux (DOY 33-51, 2018) modeled using the vd for broadleaved species (Broadleaf) and EC assessments (EC) published in Pallozzi et al. 2020. Bottom: the relative daily average wind speed (ws) and particulate matter concentration ($PM_{2.5}$).*

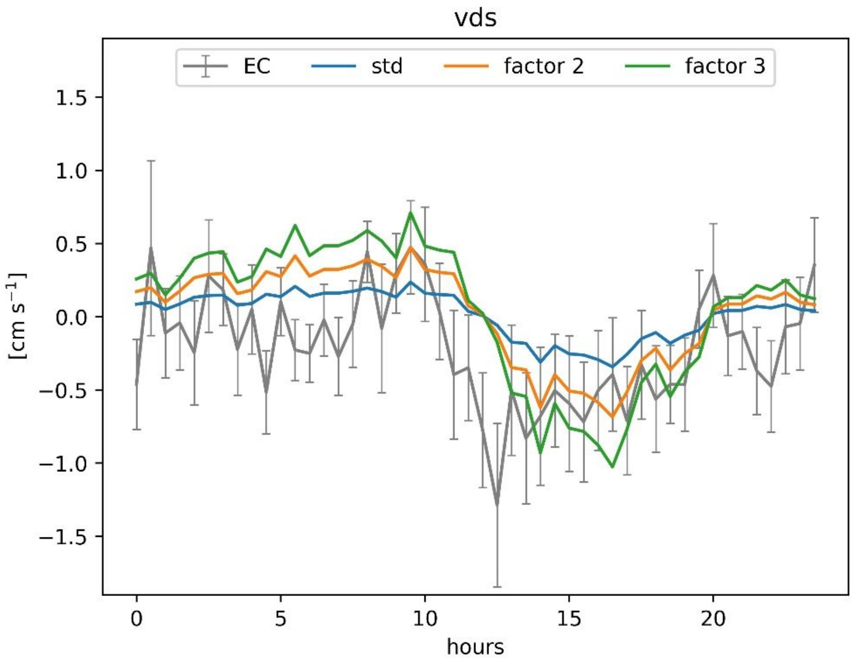

Figure S5. *Sensitivity analysis of the modeled $PM_{2.5}$ deposition velocity (vds) during the day considering a multiplicative factor of 2 and 3 of the standard parametrization (std) compared with the deposition velocity measured by eddy covariance (EC).*
